# Changes in the Microbiome in the Soil of an American Ginseng Continuous Plantation

**DOI:** 10.3389/fpls.2020.572199

**Published:** 2020-12-07

**Authors:** Jiguang Zhang, Sanhong Fan, Jun Qin, Jichen Dai, Fangjie Zhao, Liqiang Gao, Xihong Lian, Wenjing Shang, Xiangming Xu, Xiaoping Hu

**Affiliations:** ^1^State Key Laboratory of Crop Stress Biology in Arid Areas and College of Plant Protection, Northwest A&F University, Yangling, China; ^2^College of Life Science, Northwest A&F University, Yangling, China; ^3^NIAB East Malling Research (EMR), Kent, United Kingdom

**Keywords:** American ginseng, microbial diversities, high-throughput sequencing, herbal crop, continuous cropping obstacles

## Abstract

American ginseng is an important herbal medicinal crop in China. In recent years, there has been an increasing market demand for ginseng, but the production area has been shrinking due to problems associated with continuous monocropping. We analyzed the microbiome in bulk soils to assess whether and, if so, what changes in the bulk soil microbiome are associated with continuous American ginseng cropping. The alpha diversity of fungi and bacteria was significantly lower in the soils planted with American ginseng than the virgin (non-planted) land. The relative abundance of *Fusarium* spp. and *Ilyonectria* spp., known plant root pathogens, was much higher in the soils cropped with American ginseng than the non-planted. On the other hand, a number of bacteria with biodegradation function, such as *Methylibium* spp., *Sphingomonas* spp., *Variovorax* spp., and *Rubrivivax* spp., had lower abundance in the soils cropped with American ginseng than the non-cropped. In addition, soil pH was lower in the field planted with American ginseng than the non-planted. Accumulation of fungal root pathogens and reduction of soil pH may, therefore, have contributed to the problems associated with continuous monocropping of American ginseng.

## Introduction

The American ginseng (*Panax quinquefolium*), which belongs to the Araliaceae family, is a perennial herb and native to the United States and Canada. It was introduced into China in 1975 from the United States, has become one of the most used herbal medicines in Asia, and now has been grown in more than 10 provinces in China with the total planting area close to 3,700 hectares. China is the third largest production country for American ginseng following the United States and Canada, producing 3,600 tons in 2016, and it is the largest country consuming American ginseng (Wang and Wu, 2003). However, problems associated with continuous monocropping have become a major limiting factor for growing American ginseng in China (Jin et al., 2006). Soil grown with American ginseng for one season (usually 4 years) could lead to very reduced productivity on the same land for 10 years or more (Chen et al., 2012). As a result, land suitable for growing American ginseng is decreasing rapidly in China.

Problems associated with continuous monocropping occur in many crop species, such as apple, cherry, alfalfa, rice, corn, and strawberry; such problems are usually related to one or more of the following factors: deterioration of soil physicochemical properties, allelopathy/autotoxicity, soil-borne diseases, and changes in the soil microbial communities (Singh et al., 1999; Wu et al., 2008; Ying et al., 2012). Autointoxication is very common in continuous monocropping of rice, resulting in considerable yield loss (Chou, 1995). *Thielaviopsis basicola* is partially responsible for the cherry continuous cropping problem (Hoestra, 1968). *Rhizoctonia solani, Pythium intermedium*, and *Fusarium solani* are reported to play an important role in causing apple replant disease (Braun, 1995; Mazzola, 1998; Manici et al., 2003). Extracellular compounds released by *Dactylonectria torresensis* might have contributed to the severe growth reduction associated with apple replant disease (Manici et al., 2018).

Sanqi ginseng (*Panax notoginseng*) is also an important herbal plant in China. Autotoxic ginsenosides in the rhizosphere contributed to yield to a decline of Sanqi ginseng associated with continuous monocropping (Yang et al., 2015). Accumulation of soil-borne pathogen inocula and ginsenosides in Sanqi ginseng cultivated soils was considered to be one of the main reasons for replant failure in Sanqi ginseng (Li et al., 2019). Only one study was published on the continuous cropping of Asian ginseng (*Panax ginseng*), suggesting that changes in the rhizosphere microbiome due to inorganic fertilizers are one key factor resulting in the replant problem (Dong et al., 2017).

Many studies have been carried out to understand factors responsible for the replant problem in American ginseng (He et al., 2008; Bi et al., 2010), most of which have focused on toxic substances in soil. Nine phenolic compounds in the soil of American ginseng cultivation were detected and proved to inhibit radicle and shoot growth of American ginseng (Bi et al., 2010). Five groups of autotoxic compounds were found from aqueous extracts of fibrous roots of American ginseng (He et al., 2008). Adding root residue (0.02–0.5 mg/mL) to the hydroponics reduced seedling development of American ginseng and prolonged the leaf expansion period (Jiao et al., 2012).

In the present study, the main objective was to investigate the changes in soil microbial communities associated with the American ginseng production history to identify specific microbial groups related to the replant problem of American ginseng.

## Materials and Methods

### Soil Sample Collection

Soil was sampled in the spring of 2017 from three fields (Zakoushi, Yingpan, and Miaotaizi) of Liuba county, Shaanxi province, China. The area of the fields varied from ca. 600 to 1,200 m^2^. At Zakoushi, the fields were grown with American ginseng for 0–4 years, coded as LZCK for control (year 0) and LZ1, LZ2, LZ3, and LZ4 for years 1–4 years, respectively. At the Yingpan and Miaotaizi fields, American ginseng has been grown for 8 and 12 years; samples were coded as LZA2 for Yingpan and LZA3 for Miaotaizi with LZA2CK and LZA3CK as the respective controls (non-ginseng planted soil) (Table 1). In all the fields, American ginseng was grown with standard cultural practices. Briefly, the soil was fertilized with the humus of a primitive forest and plowed to the depth of ca. 30 cm prior to planting. American ginseng is planted in March and harvested in October of the fourth year. A total of 31 soil samples were collected from the fields with varying planting years (Table 1). For each sample, soil was collected from the top layer (0–20 cm) with a soil core sampler (2.5 cm in diameter) at five locations. Then, the soils were blended thoroughly and put into in sterile polythene bags. After the samples were transported to the laboratory, soils were immediately divided into two parts: one part was stored at −80°C until further use, and the other part was air-dried at room temperature for analysis of physiochemical properties.

**Table 1:** Details of soil sample taken from three fields (Zakoushi, Yingpan, and Miaotaizi) with different cropping history of American ginseng in Liuba county, Shaanxi province, China.

**Sample location**	**Longitude/** **Latitude**	**Code of samples**	**Cultivation years**	**Number of samples**
Zakoushi field	106°43′13′′E/ 33°38′41′′N	LZ1	1	3
		LZ2	2	3
		LZ3	3	3
		LZ4	4	5
		LZCK	0	3
Yingpan field	106°43′16′′E/ 33°37′41′′N	LZA2	8	5
		LZA2CK	0	3
Miaotaizi field	106°84′E/ 33°68′N	LZA3	12	3
		LZA3CK	0	3
Total				31

### Soil DNA Isolation and Sequencing

Total soil DNA was extracted with the NucleoSpin Soil Kit (Macherey-Nagel, Germany) following the manufacturer's instructions. The V4 region of bacterial 16s rDNA and the ITS1 region of fungal rDNA were amplified with the universal primer pairs 515F/806R (515F: GTGCCAGCMGCCGCGGTAA and 806R: GGACTACHVGGGTWTCTAAT) (Caporaso et al., 2011) and ITS1/ITS2 (ITS1: CTTGGTCATTTAGAGGAAGTAA and ITS2: GCTGCGTTCTTCATCGATGC) (Walters et al., 2016), respectively. Both forward and reverse primers were tagged with the Illumina adaptor and index sequences. PCR reactions were performed with the Phusion High-Fidelity PCR Master Mix (NEB, USA) in a 50-μL reaction, including 300 mM of each primer, 30 ng template DNA, and 25 μL PCR Master Mix. The PCR condition was as follows: predenaturation at 94°C for 3 min, 30 cycles of 94°C for 30 s, 56°C (V4)/55°C (ITS1) for 45 s, 72°C for 45 s, and final extension at 72°C for 10 min. PCR products from different samples were pooled together (equal volume) and purified with AGENCOURT AmpureXP beads (Beckman Coulter, China) as a library. The library was quantified with the Agilent 2100 bioanalyzer and ABI StepOnePlus Real-time PCR system. Finally, the validated library was sequenced on the Illumina MiSeq platform by BGI (Shenzhen, China) to generate 250 bp paired-end reads.

### Sequence Processing

Raw sequences were preprocessed to obtain clean sequence reads with the in-house pipeline of BGI Co., Ltd. Paired-end reads were then merged using fast length adjustment of short (FLASH) reads (v1.2.11). Minimal overlapping length was 15 bp with the maximum mismatching ratio in the overlapped region set to 0.1. Unique sequences with only one read were discarded. Then, all unique sequences were clustered into operational taxonomic units (OTUs) with USEARCH v7.0.1090 at 97% similarity with a representative sequence generated for each OTU. The SINTAX algorithm (https://www.drive5.com/usearch/manual/sintax_algo.html) then assigned each OTU sequence to taxonomic ranks by aligning the representative sequence against the Ribosomal Database Project (RDP) Classifier v.2.2 trained on the bacterial database Greengene v201305 (Cole et al., 2014) and the UNITE_v7.2 fungal database (Kõljalg et al., 2013). A confidence value of 0.6 was used as the cutoff when assigning an OTU to a specific taxonomy group. Then, an OTU table was generated by aligning all sequences filtered with far less stringent criteria with the OTU representative sequences as described previously (Deakin et al., 2018). All OTU processing was carried out with the UPARSE pipeline (v10.0) (Edgar, 2013).

### Data Analysis

The original OTU tables were normalized (rarefication) with Qiime2 for all subsequent statistical analysis; the sampling depths were 57,000 and 42,000 for fungi and bacteria, respectively.

We conducted two types of analysis. First, we analyzed the effect of cropping years (0–4 years) at Zakoushi through a one-way ANOVA. Second, we compared microbial communities between the virgin land and the soils grown with American ginseng for multiple years across the three fields. In this analysis, samples of the fourth year (LZ4) were chosen at Zakoushi field to compare with other two fields, and the three fields (sites) were treated as a blocking factor; namely each field (site) represents a random sample of planting vs. non-planting comparisons. Thus, the statistical model is of the form: “Fields + Planting vs. Non-planting.”

Alpha diversity indices (Shannon, Chao1, and Robbins indices and observed feature) were calculated. The non-parametric methods of the Kruskal–Wallis test and trimmed means method were applied to assess the year effect at Zakoushi and planting effect across the three sites, respectively. To assess the differences in the overall microbial communities among samples, Bray–Curtis indices between samples were calculated and were subjected to non-metric multidimensional scaling (NMDS); the effects of treatments (planting years or planting vs. non-planting) were analyzed with the Adonis method through PERMANOVA (as implemented in R package vegan 2.3-1). The differences in fungal or bacterial relative abundance aggregated at each taxonomic level (with RDP Classifier v2.2 at the confidence of 0.6) between soils planted with and without American ginseng were analyzed with the compare group function in R package metacoder v0.3.3 (Foster et al., 2017) with *p*-values adjusted with the Benjamini–Hochberg method. The metacoder package was also used to display tree view graphs of this differential abundance analysis.

### Measuring Soil pH and Nutrient Elements

Soil pH was measured for all individual samples with 1 mol (M) KCl as suspending media to avoid seasonal variability (Collins et al., 1970). Each soil sample was air-dried at room temperature for 3 days, ground thoroughly, and passed through 1-mm sieve. A subsample of 8.0 g soil was dissolved into 20.0 ml 1M KCl in a 50-ml beaker, mixed with a glass rod, and then kept at room temperature for 30 min for pH measurement with a pH meter (Mettler FE20 FiveEasy Plus™ pH, Germany). The standard pH buffer solutions of 4.01, 6.87, and 9.18 were used to adjust the pH meter. Available potassium was estimated with the flame photometric method (M410, Sherwood Scientific Ltd., Cambridge, UK). Available phosphorus was estimated with the spectrophotometric method (Shimadzu UV2450, Shimadzu Scientific Instrument, Kyoto, Japan).

## Results

### General Characteristics of Sequence Data

A total of 2,372,515 ITS sequences and 1,843,202 16S sequences were obtained from the 31 samples. The number of raw sequence reads per sample ranged from 78,729 to 83,757 for ITS and from 60,740 to 63,348 for 16S. The number of sequences per sample after quality filtering ranged from 72,492 to 78,934 (ITS) and from 58,430 to 60,313 (16S) per sample. The number of OTUs in each sample ranged from 383 to 877 (fungi) and from 1,976 to 4,088 (bacteria). Sequencing depth is sufficient for all samples (Supplementary Figure 1). Raw sequences were uploaded to the NCBI BioProject database (Accession: PRJNA640251).

### Fungal Diversity

**Figure 1:**
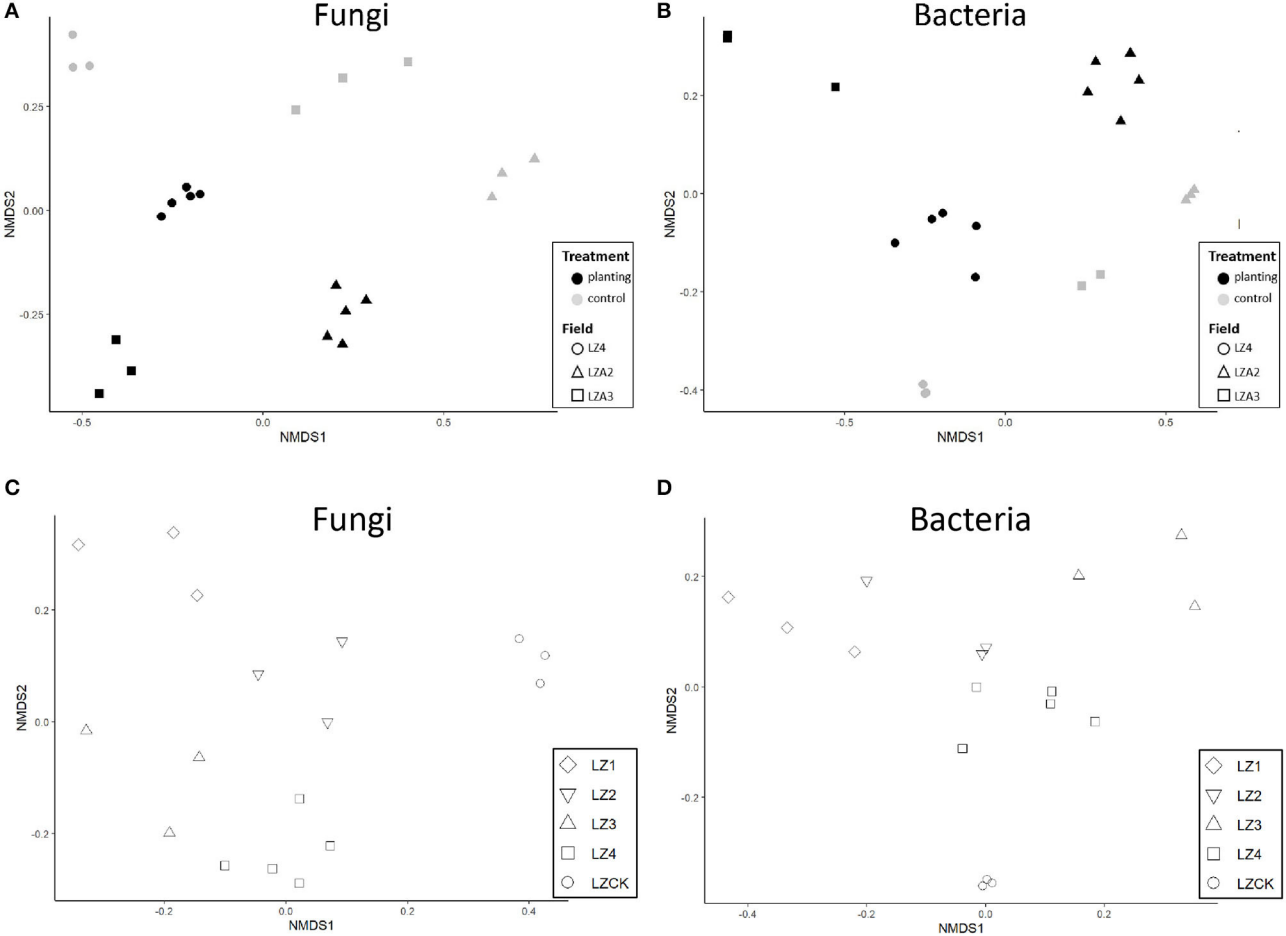
Non-metric multidimensional scaling (NMDS) of rarefied OTU data for fungi and bacteria in soil samples. **(A)** Fungi in different fields. **(B)** Bacteria in different fields. **(C)** Fungi in samples with 0–4 years of cropping with American ginseng at Zakoushi. **(D)** Bacteria in samples with 0–4 years of cropping with American ginseng at Zakoushi.

The alpha diversity indices (Shannon, observed feature, and Chao1) showed that fungal alpha diversity decreased (*P* < 0.01) in the soils planted with American ginseng compared to the non-planted, but there were no significant differences in the Robbins indices. Adonis analysis of the Bray–Curtis indices indicated significant (*P* < 0.001) differences between soils grown with American ginseng for multiple years and the virgin land. NMDS showed that samples from soils grown with American ginseng for multiple years were clearly separated from those virgin lands (Figure 1A); as expected, there were large differences between the three fields as well. There were clear separations between samples from the fields grown with American ginseng for 0–4 years at Zakoushi (Figure 1C). Multiple comparison based on PERMANOVA also showed that the overall fungal community from the field grown with the ginseng for 4 years (LZ4) differed (*P* < 0.05) from LZCK (control), LZ1, LZ2, and LZ3.

**Figure 2:**
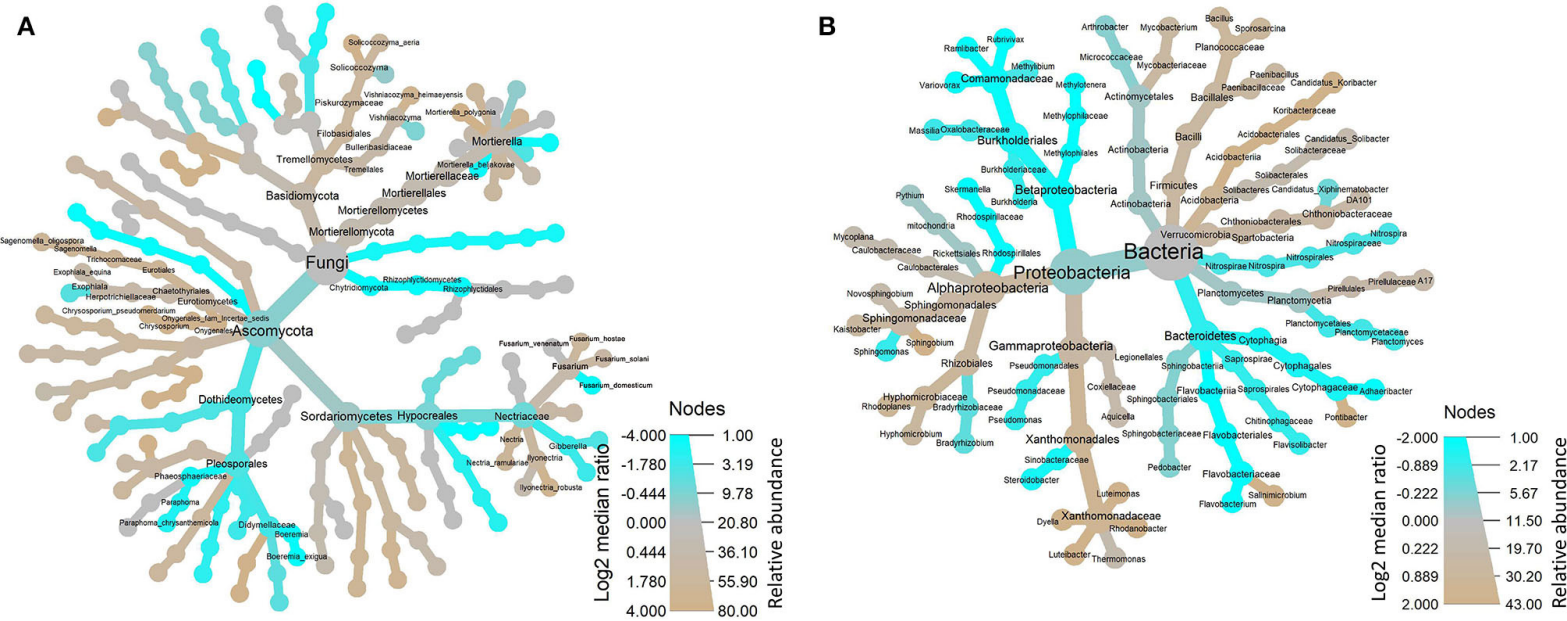
Tree views of the relative abundance of fungi **(A)** and bacteria **(B)** at several taxonomic ranks in the soil grown with and without American ginseng.

The OTUs with taxon for fungi were finally counted as 80 (Supplementary Table 1) not including those whose OTUs were identified as a taxon named unidentified in the database, those whose OTUs were confirmed but could not be found in the database, and those whose relative abundance was <0.05% in all 31 samples. The tree view in Figure 2A was constructed using these data. At the class rank, the abundance of Dothideomycetes fungi decreased in the soils planted with American ginseng, and Eurotiomycetes increased compared to non-planted soils. At the order rank, two orders (Eurotiales and Pleosporales) increased significantly in the soils grown with American ginseng compared to the non-planted. At the family rank, Trichocomaceae had an increased relative abundance, whereas Phaeosphaeriaceae and Didymellacea had decreased relative abundance. At the genus rank, *Sagenomella* had an increased relative abundance, and *Boeremia* and *Gibberella* had decreased relative abundance. At the species rank, only *Boeremia exigua* and *Paraphoma chrysanthemicola* had decreased relative abundance, whereas eight species, including known plant root pathogens (*Ilyonectria robusta, Vishniacozyma heimaeyensis*, and *Fusarium hostae*) of American ginseng, had increased relative abundance in the ginseng-cropping compared to non-planted soils.

*Mortierella* spp. was the dominant fungal species in all soil samples, and its relative abundance varied from 22.2 to 52.6% (Supplementary Tables 2, 3). *Fusarium* spp. was not detected in any control soil samples but was found in all soil samples from the ginseng-grown soils at Zakoushi. The relative abundance of *Fusarium* spp. increased with the cropping years, accounting for 1.4, 2.0, 2.2, and 3.2% in the first, second, third, and fourth years, respectively (Supplementary Table 2).

### Bacterial Diversity

The bacterial alpha diversity decreased greatly in the fields grown with American ginseng for multiple years compared to the virgin lands. All the alpha diversity indices calculated showed significant differences: Shannon (*P* < 0.01), Chao1 (*P* < 0.05), Robbins (*P* < 0.05), and observed feature (*P* < 0.001). Similarly, the beta-diversity indices (Bray–Curtis indices) also differed (*P* < 0.001) between the soils grown with and without America ginseng across the three fields as shown by the Adonis analysis. NMDS analysis showed a clear separation of samples between the soils grown with and without ginseng at each field (Figure 1B). Similarly, samples from the soils grown with ginseng for 0–4 years at Zakoushi were also separated (Figure 1D). The overall bacterial community in the soils grown with ginseng for 4 years differed (*P* < 0.05) from all other samples at Zakoushi.

**Figure 3:**
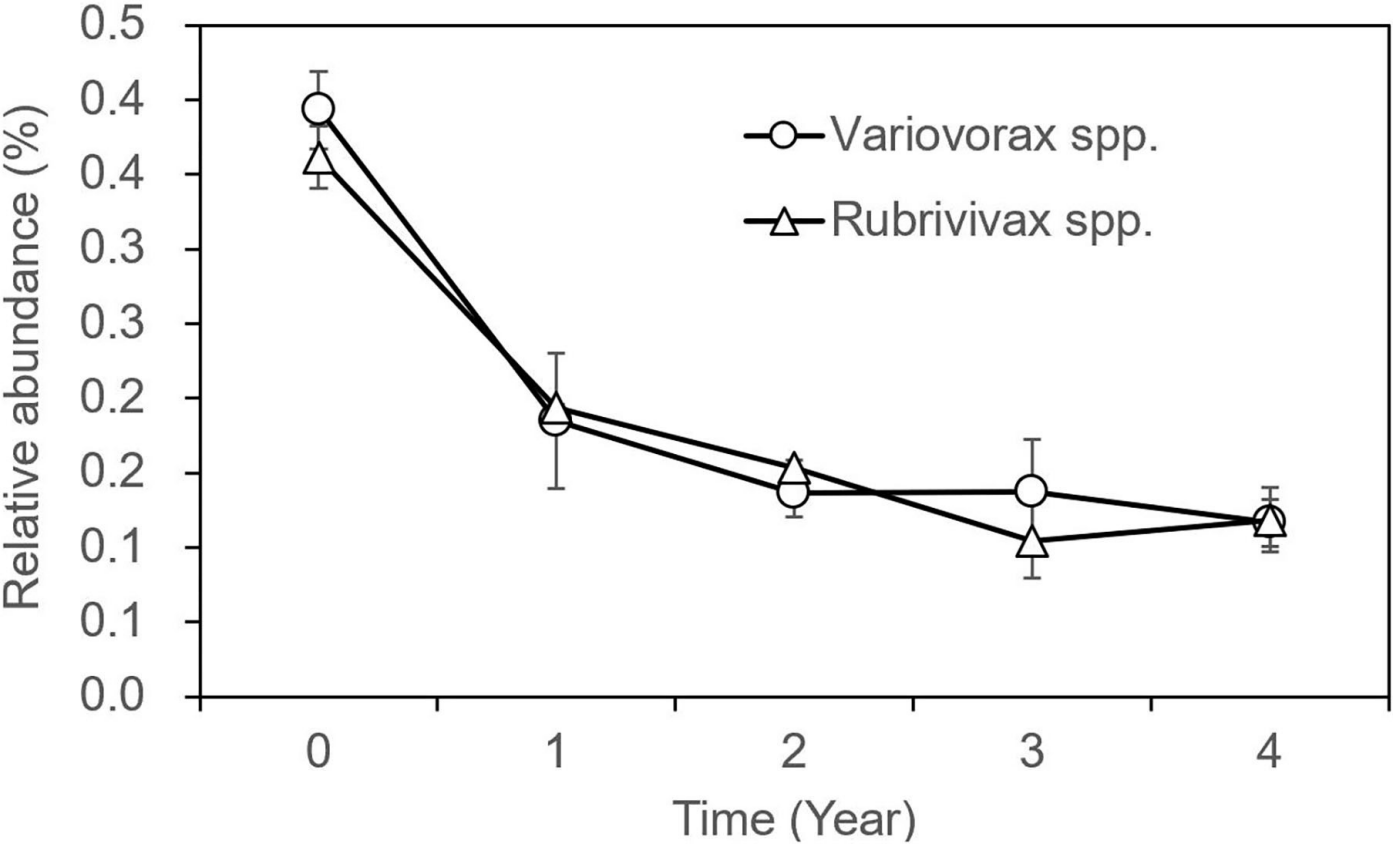
The variation of relative abundance in two bacterial genera in the soil grown with American ginseng for 0–4 years. Open circle, the OTUs of *Variovoras* spp. Open triangle, the OTUs of *Rubrivivax* spp.

**Figure 4:**
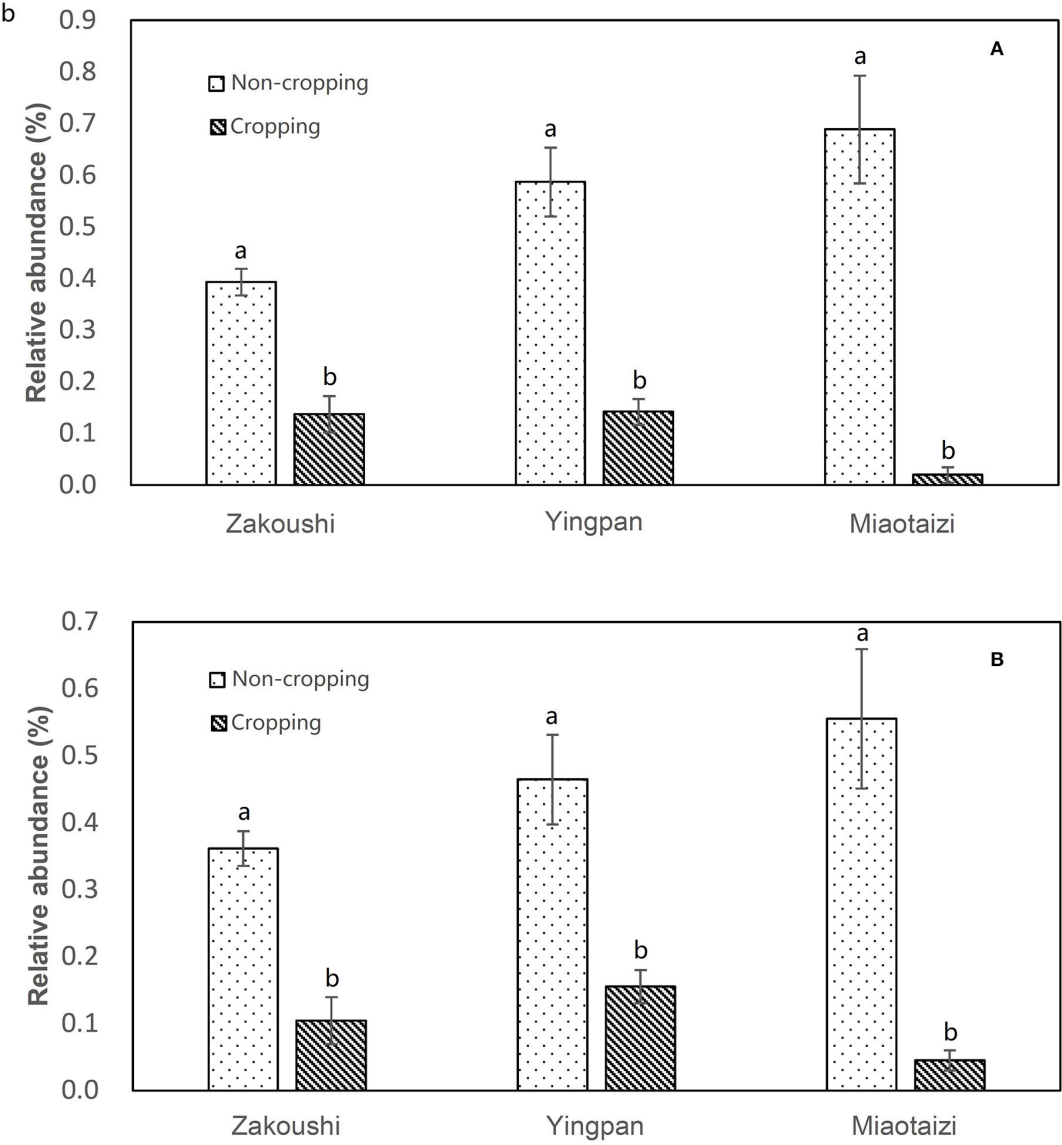
Relative abundance of *Variovorax* spp. **(A)** and *Rubrivivax* spp. **(B)** in the soil from three fields. Different letters represent statistically significant differences (*p* < 0.05).

The OTUs with taxon for bacteria were finally counted as 43 (Supplementary Table 4), not including those whose OTUs were identified as a taxon named unidentified in the database, those whose OTUs were confirmed but could not be found in the database, and those whose relative abundance was <0.05% in all 31 samples. The tree view in Figure 2B was constructed using these data. As shown in Figure 2B, at the phylum rank, three phyla (Betaproteobacteria, Nitrospirae, and Bacteroidetes) decreased in the soil planted with American ginseng, and four phyla (Verrucomicrobia, Acidobacteria, Firmicutes, and Alphaproteobacteria) increased compared to non-planted soils. At the class rank, the relative abundance of Solibacteres, Acidobacteriia, and Bacilli was higher in the soils planted with American ginseng than non-planted soils. At the order rank, five orders (Methylophilales, Burkholderiales, Saprospirales, Nitrospirales, and Pseudomonadales) had decreased relative abundance in the soils grown with American ginseng compared to non-planted soils. At the genus level, 13 genera decreased, whereas six genera grew in relative abundances. Most of the decreased genera, i.e., *Methylibium, Rubrivivax, Variovorax*, and *Sphingomonas*, were involved in the degradation of certain compound substances, such as diphenyl ether, isoproturon, and atrazine. Interestingly, an alkali-tolerant bacterial genus, *Ramlibacter*, was found decreased, and one phylum, Acidobacteria, which are suitable for growing in acidic conditions, were found to be higher in the soils grown with American ginseng than non-planted soils. This coincides with the decreasing soil pH in the soil grown with American ginseng. Among the increasing genera, Xanthomonadaceae was proved to be a plant pathogen, and *Sphingbium* was involved in the degradation of phenol, which was one of the compounds inhibiting radicle and shoot growth of American ginseng. The relative abundance of two bacteria, *Variovorax* spp. and *Rubrivivax* spp., decreased with the increase of cropping years (Figure 3). The amount of *Variovorax* spp. declined from 0.39% in non-planting soil to 0.14% in planting soil at Zakoushi, dropped from 0.59 to 0.14% at Yingpan, and dropped from 0.69 to 0.02% at Miaotaizi (Figure 4A). The abundance of *Rubrivivax* spp. decreased from 0.36 to 0.14% at Zakoushi, dropped from 0.46 to 0.16% at Yingpan, and fell from 0.56 to 0.05% at Miaotaizi (Figure 4B).

### Changes of pH value and Concentration of Potassium and Phosphorus Elements in Soil

**Figure 5:**
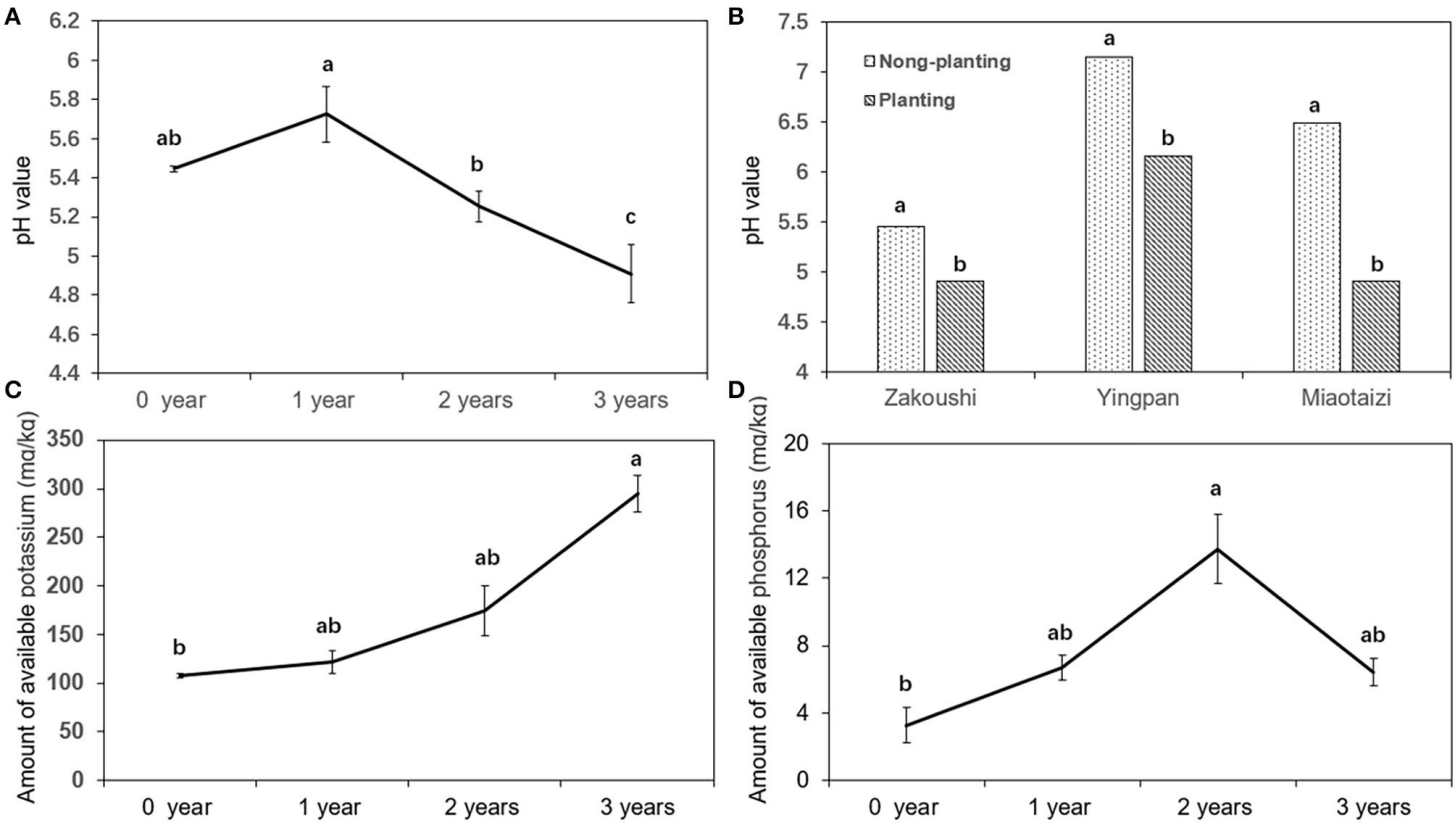
Change of soil pH and nutrient elements in the soil grown with American ginseng for various lengths. **(A)** Soil pH in samples with 0–4 years of cropping with American ginseng at Zakoushi. **(B)** Comparison of soil pH in the soil planted with and without American ginseng in three fields. **(C)** Available potassium in samples with 0–4 years of cropping with American ginseng at Zakoushi. **(D)** Available phosphorus in samples with 0–4 years of cropping with American ginseng at Zakoushi. Different letters represent statistically significant differences (*p* < 0.05).

The soil pH decreased with the increasing year of cropping with American ginseng at Zakoushi (Figure 5A). At the other two fields (LZA2 and LZA3), soil pH was also reduced (*P* < 0.05) from 7.2 (control) to 6.2 at Yingpan and decreased from 6.5 (control) to 4.9 at Miaotaizi (Figure 5B). The available potassium increased (*P* < 0.05) with the continuous planting years (Figure 5C), but there was no linear trend in the available phosphorus (Figure 5D).

## Discussion

Soil sickness is a phenomenon that causes the reduction in both crop yield and quality when the same crop or its relative species are cultivated on the same soil successively (Yu, 2008). Cultivation of American ginseng for one crop season usually takes 4 years in the soil until harvesting. Through analysis of soils grown with American ginseng for a number of years, we identified several possible causes that could contribute to the replant issue in American ginseng: (1) accumulation of several plant fungal root pathogens (e.g., *Fusarium oxysporum, F. solani, F. proliferatum*, and *Gibberella baccata*); (2) reduction of some potential beneficial bacteria, such as *Methylibium* spp., *Sphingomonas* spp., *Variovorax* spp., and *Rubrivivax* spp.; and (3) gradual decreasing soil pH value.

*Fusarium* spp. is a damaging plant pathogen, infecting many plant species and causing root rot and wilt diseases (Huang et al., 1998; Bi et al., 2011). In the diseased roots of American ginseng, *F. oxysporium* and *F. solani* were commonly isolated (data not shown). Punja (1997) isolated *Fusarium solani, F. oxysporum, F. avenaceum*, and *F. equiseti* from the diseased roots of American ginseng. *Fusarium solani, F. oxysporum, F. tricinctum, F. proliferatum*, and *Cylindrocarpon destructans* were all isolated from the diseased roots of American ginseng, and among these, *F. solani* and *F. oxysporum* were mainly responsible for root rot diseases (Bi et al., 2011). The present finding agrees with those previous findings as the relative abundance of *Fusarium* spp. in the soil grown with American ginseng increased considerably. The present study also suggests other common root pathogens in the *Nectriaceae* family may also contribute to the replant issue of American ginseng, including *Ilyonectria_robusta, Dactylonectria anthuriicola*, and *I. mors-panacis*, which causes red-skin root in *Panax ginsen*g (Lu et al., 2019). Therefore, accumulation of pathogens is likely to be one of the reasons for the reduced productivity of American ginseng in continuous monocropping.

*Mortierella* spp. has been widely used in biosynthesis. Certik and Shimizu (1999) report that arachidonic acid, dihomo-g-linolenic acid, and mead acid are produced by *Mortierella* spp. In the present study, *Mortierella* spp. is the dominant fungal group in all soil samples. Although, Lu et al. (2014) isolated *Mortierella* sp. from the roots of red-skin Asian ginseng (*P. ginseng*), it has not yet been demonstrated whether *Mortierella* spp. is responsible for red-skin disease. As *Mortierella* spp. constituted such a high proportion in all the ginseng soil, further research is needed to ascertain whether ginseng health is affected by *Mortierella* spp.

In the soils grown with American ginseng for 1–4 years, the abundance of two genera, *Variovorax* spp. and *Rubrivivax* spp., declined with increasing cropping years. Some species of *Variovorax* are able to metabolize a large variety of different substrates (Satola et al., 2013). *V. paradoxus* showed great capabilities in increasing the root and shoot biomass of pea and the uptake of N, P, K, Ca, and Mg (Jiang et al., 2012) and degrading lots of compounds, such as isoproturon (Hussain et al., 2011), linuron (Sorensen et al., 2005), atrazine (Smith et al., 2005), polycyclic aromatic hydrocarbons (Eriksson et al., 2003; Young et al., 2006), chlorinated hydrocarbons (Humphries et al., 2005), and methyl tertiary butyl ether (Zaitsev et al., 2007). *Rubrivivax gelatinosus* can degrade pollutants in fish industry effluent (Leandro et al., 2011). Hence, we speculate that *Variovorax* spp. and *Rubrivivax* spp. may play a role in degrading toxicants in the soil growing with American ginseng for multiple years. Their decrease might lead to accumulation of some toxic substances, which might impact negatively on American ginseng growth. Further research is needed to support or disprove our speculation.

Soil physicochemical property deterioration is believed to be a factor responsible for reduced cropping potentials in the continuous cropping of *P. ginseng*. Specific gravity and bulk density of the soils grown with ginseng increased, and soil porosity decreased compared to the soil without ginseng (Laura et al., 2000; Wu et al., 2008). reports that low pH restricts nitrification rates and increases concentrations of certain elements known to be toxic to many plants (e.g., aluminum). Soil pH affected root length, plant height, nodule, and pod number of cowpea (Joe and Allen, 1980). Our results show that the pH value in American ginseng cropping soil declined significantly from 5.72 in the first year to 4.91 in the third year at Zakoushi, from 7.16 to 6.16 in the soil at Yingpan, and from 6.49 to 4.92 in the soil at Miaotaizi. The soil pH 4.91 is outside the pH range of 5.5–6.0 suitable for American ginseng (Hong, 1978). Interestingly, the present study revealed that the relative abundance of an alkali-tolerant bacterium, *Ramlibacter*, decreased, but the phylum of Acidobacteria increased in the soils grown with American ginseng. Low pH (and associated changes in specific microorganisms in the soil) may be another factor contributing to reduced growth in continuous monocropping of American ginseng.

There are other factors that could contribute to reduced crop productivity in monocropping. Root exudates can lead to changes in soil physicochemical properties and the microbial community; the microbial community may alter the degradation of root exudates and further affect the physicochemical properties of the soil; the change of soil physicochemical properties can, in turn, influence microbial community structures. Further research is needed to study the cross-talk among the microbial community, root exudates, and soil physicochemical properties in American ginseng monocropping.

## Data Availability Statement

Raw sequences were uploaded to the NCBI BioProject database (Accession: PRJNA640251).

## Author Contributions

XH, XX, and SF planned and designed the research. JZ, FZ, JQ, WS, JD, SF, and XL performed the experiments. SF, JD, JZ, and XH analyzed the data. JZ, JQ, XX, and XH wrote the manuscript. All authors contributed to the article and approved the submitted version.

## Conflict of Interest

The authors declare that the research was conducted in the absence of any commercial or financial relationships that could be construed as a potential conflict of interest.

## Supplementary Material

Supplementary Figure 1 Number of sequences plotted against the coverage of OTUs; each line is standard for one of the 31 samples.

Supplementary Table 1 The OTU number with taxon for fungi in the soil of different American ginseng cropping years.

Supplementary Table 2 Fungal relative abundance in American ginseng cropped soil for 0–4 years.

Supplementary Table 3 Relative abundance of fungi in the soil planted and non-planted with American ginseng at Yinpan and Miaotaizi.

Supplementary Table 4 The OTU number with taxon for bacteria in the soil of different American ginseng cropping years.
