# Supplementary material for: Changes in the Microbiome in the Soil of an American Ginseng Continuous Plantation
Source: Front Plant Sci. 2020 Dec 7;11:572199. doi: 10.3389/fpls.2020.572199 (PMC7750500; doi:10.3389/fpls.2020.572199)
Supplement: Supplementary Figure 1 — Number of sequences plotted against the coverage of OTUs; each line is standard for one of the 31 samples. [file Data_Sheet_1.zip › Table 2 - 2020-12-02T125505.212.DOCX]

**TABLE S1** The OTUs number with taxon for fungi in the soil of different American ginseng cropping years.

| **Fungal name** | **OTUs number** | | | | | | | | |
| --- | --- | --- | --- | --- | --- | --- | --- | --- | --- |
|  | LZ1 | LZ2 | LZ3 | LZ4 | LZCK | LZA2 | LZA2CK | LZA3 | LZA3CK |
| *Acremonium furcatum* | 0 | 12 | 56 | 79 | 44 | 176 | 574 | 53 | 1204 |
| *Alternaria brassicae* | 0 | 0 | 0 | 0 | 457 | 0 | 0 | 0 | 0 |
| *Atractospora reticulata* | 61 | 320 | 445 | 813 | 352 | 8 | 1 | 2 | 2 |
| *Basidiobolus ranarum* | 0 | 0 | 77 | 435 | 0 | 0 | 0 | 0 | 0 |
| *Boeremia exigua* | 121 | 469 | 8 | 7 | 406 | 196 | 4840 | 5 | 2206 |
| *Cadophora luteo-olivacea* | 21 | 3 | 415 | 1194 | 19 | 2 | 0 | 247 | 0 |
| *Calonectria asiatica* | 67 | 132 | 722 | 165 | 377 | 0 | 0 | 2 | 0 |
| *Chrysosporium pseudomerdarium* | 28 | 2 | 280 | 546 | 1 | 207 | 29 | 107 | 13 |
| *Cladosporium delicatulum* | 22776 | 3900 | 10480 | 1842 | 6476 | 2354 | 1954 | 1278 | 1794 |
| *Cladosporium ramotenellum* | 1059 | 738 | 1124 | 10 | 320 | 367 | 49 | 125 | 12 |
| *Clonostachys rosea* | 138 | 242 | 64 | 129 | 479 | 54 | 1599 | 2249 | 737 |
| *Coprinellus bisporus* | 26 | 2506 | 2 | 193 | 0 | 161 | 0 | 1 | 44 |
| *Corynespora cassiicola* | 20 | 130 | 0 | 159 | 54 | 85 | 9662 | 162 | 310 |
| *Cystofilobasidium macerans* | 88 | 92 | 727 | 224 | 7249 | 28 | 59 | 591 | 1458 |
| *Entoloma graphitipes f. cystidiatum* | 0 | 0 | 1 | 2 | 0 | 0 | 7149 | 0 | 2 |
| *Entoloma llimonae* | 21 | 546 | 8 | 3 | 11 | 67 | 8 | 2 | 0 |
| *Epicoccum nigrum* | 1179 | 895 | 2150 | 5822 | 14028 | 7549 | 6047 | 204 | 1433 |
| *Exophiala equina* | 3002 | 2876 | 4703 | 4505 | 1261 | 1903 | 239 | 2280 | 881 |
| *Exophiala opportunistica* | 80 | 75 | 1 | 13 | 573 | 57 | 36 | 0 | 10 |
| *Fusarium domesticum* | 13 | 2 | 198 | 17 | 0 | 112 | 1896 | 97 | 1176 |
| *Fusarium hostae* | 1009 | 392 | 668 | 643 | 658 | 3774 | 558 | 931 | 283 |
| *Fusarium solani* | 763 | 517 | 2746 | 843 | 98 | 3090 | 1348 | 5565 | 1753 |
| *Fusarium venenatum* | 0 | 0 | 0 | 0 | 0 | 0 | 446 | 0 | 0 |
| *Fusicolla aquaeductuum* | 292 | 522 | 354 | 472 | 513 | 1067 | 148 | 201 | 373 |
| *Gibberella baccata* | 1520 | 4915 | 1496 | 2633 | 8070 | 1041 | 1314 | 615 | 2145 |
| *Gibberella intricans* | 476 | 773 | 3 | 436 | 689 | 3846 | 16350 | 222 | 2699 |
| *Guehomyces pullulans* | 536 | 8721 | 2352 | 9364 | 4720 | 3473 | 2340 | 2198 | 1977 |
| *Herpotrichia juniperi* | 17 | 412 | 84 | 335 | 285 | 26 | 384 | 5 | 32 |
| *Humicola nigrescens* | 1971 | 915 | 847 | 2203 | 815 | 718 | 1471 | 2047 | 1286 |
| *Hymenula cerealis* | 82 | 20 | 10 | 59 | 941 | 6 | 0 | 1 | 49 |
| *Ilyonectria macrodidyma* | 2029 | 2026 | 7429 | 2219 | 276 | 5899 | 2704 | 344 | 1514 |
| *Ilyonectria robusta* | 69 | 81 | 496 | 48 | 40 | 431 | 40 | 957 | 11 |
| *Itersonilia perplexans* | 6 | 0 | 2 | 28 | 1732 | 209 | 1 | 31 | 0 |
| *Kernia nitida* | 0 | 13 | 34 | 895 | 1 | 79 | 298 | 0 | 15 |
| *Lectera colletotrichoides* | 0 | 0 | 0 | 0 | 0 | 1 | 15728 | 0 | 5 |
| *Leptosphaeria sclerotioides* | 239 | 1810 | 250 | 4318 | 225 | 743 | 206 | 0 | 218 |
| *Metarhizium marquandii* | 324 | 550 | 491 | 339 | 70 | 157 | 524 | 12 | 757 |
| *Minimedusa polyspora* | 207 | 996 | 169 | 1887 | 3232 | 380 | 0 | 289 | 966 |
| *Mortierella alpina* | 2181 | 6320 | 2556 | 4126 | 1614 | 11081 | 27036 | 2209 | 17100 |
| *Mortierella amoeboidea* | 518 | 792 | 1377 | 913 | 449 | 1340 | 2593 | 496 | 478 |
| *Mortierella antarctica* | 159 | 150 | 61 | 110 | 27 | 2129 | 2035 | 0 | 0 |
| *Mortierella beljakovae* | 704 | 0 | 0 | 9 | 58 | 0 | 49 | 0 | 38 |
| *Mortierella bisporalis* | 223 | 237 | 332 | 275 | 288 | 1183 | 12 | 10 | 1232 |
| *Mortierella elongata* | 1178 | 778 | 104 | 1476 | 4 | 39602 | 4593 | 1367 | 9207 |
| *Mortierella exigua* | 4753 | 3699 | 1043 | 4661 | 2521 | 12077 | 2853 | 308 | 7595 |
| *Mortierella gamsii* | 216 | 404 | 1953 | 713 | 119 | 23 | 244 | 2 | 987 |
| *Mortierella hyalina* | 2456 | 5783 | 1003 | 20297 | 2504 | 166 | 562 | 7903 | 576 |
| *Mortierella minutissima* | 3970 | 3526 | 1249 | 3666 | 523 | 4097 | 1061 | 157 | 1578 |
| *Mortierella paraensis* | 428 | 4 | 3 | 0 | 41 | 0 | 0 | 0 | 0 |
| *Mortierella polygonia* | 0 | 4087 | 327 | 2098 | 0 | 3448 | 522 | 381 | 668 |
| *Mortierella pseudozygospora* | 187 | 213 | 162 | 918 | 651 | 49 | 0 | 20 | 0 |
| *Mortierella sclerotiella* | 22 | 1032 | 0 | 0 | 142 | 0 | 0 | 0 | 0 |
| *Mrakia aquatica* | 18 | 16 | 22 | 18 | 2743 | 80 | 19 | 2 | 100 |
| *Nectria ramulariae* | 167 | 322 | 326 | 1055 | 456 | 1261 | 196 | 168 | 117 |
| *Olpidium brassicae* | 92 | 364 | 73 | 1642 | 173 | 272 | 2965 | 2 | 393 |
| *Paraboeremia selaginellae* | 88 | 894 | 41 | 304 | 0 | 77 | 262 | 18 | 2 |
| *Paraphoma chrysanthemicola* | 1023 | 2271 | 310 | 1308 | 3025 | 190 | 652 | 1 | 1182 |
| *Phaeosphaeria fuckelii* | 0 | 0 | 0 | 0 | 0 | 0 | 0 | 0 | 8511 |
| *Plectosphaerella cucumerina* | 35 | 86 | 135 | 206 | 167 | 3089 | 2692 | 1181 | 809 |
| *Preussia alloiomera* | 53 | 404 | 201 | 135 | 5 | 159 | 76 | 0 | 0 |
| *Preussia flanaganii* | 829 | 137 | 185 | 585 | 113 | 1489 | 1663 | 33 | 224 |
| *Preussia pilosella* | 18 | 68 | 18 | 21 | 5 | 483 | 149 | 10 | 70 |
| *Pseudogymnoascus roseus* | 51 | 63 | 613 | 1011 | 197 | 191 | 160 | 260 | 178 |
| *Pyronema domesticum* | 0 | 0 | 0 | 0 | 0 | 107 | 0 | 0 | 1192 |
| *Rhizophlyctis rosea* | 668 | 0 | 5 | 220 | 0 | 0 | 5 | 0 | 147 |
| *Sagenomella oligospora* | 15 | 229 | 439 | 911 | 66 | 1484 | 272 | 453 | 539 |
| *Scutellinia scutellata* | 23 | 283 | 26 | 159 | 7 | 379 | 726 | 67 | 108 |
| *Solicoccozyma aeria* | 1875 | 2828 | 2925 | 6416 | 1603 | 13773 | 1784 | 1398 | 2999 |
| *Solicoccozyma terrea* | 211 | 947 | 1118 | 1588 | 3217 | 494 | 26 | 1345 | 1194 |
| *Solicoccozyma terricola* | 593 | 3729 | 8703 | 14187 | 7046 | 39 | 3 | 1711 | 284 |
| *Sonoraphlyctis ranzonii* | 0 | 4 | 0 | 0 | 1387 | 0 | 0 | 0 | 0 |
| *Stachybotrys microspora* | 0 | 0 | 0 | 0 | 0 | 0 | 0 | 3 | 501 |
| *Tetracladium breve* | 836 | 2639 | 2301 | 8593 | 1975 | 6260 | 1183 | 1695 | 2386 |
| *Tetracladium marchalianum* | 388 | 5884 | 1261 | 2645 | 6657 | 1934 | 1315 | 552 | 318 |
| *Thelebolus globosus* | 141 | 503 | 219 | 2424 | 30 | 4608 | 2707 | 0 | 268 |
| *Trichocladium opacum* | 2705 | 3541 | 1724 | 7997 | 2482 | 1988 | 148 | 233 | 257 |
| *Typhula variabilis* | 0 | 1484 | 587 | 1237 | 206 | 1069 | 10 | 170 | 43 |
| *Vishniacozyma heimaeyensis* | 58 | 58 | 1197 | 250 | 13 | 83 | 42 | 1949 | 54 |
| *Vishniacozyma victoriae* | 250 | 72 | 33 | 194 | 2246 | 544 | 102 | 1 | 134 |
| *Waitea circinata* | 0 | 1 | 4 | 0 | 37 | 248 | 0 | 90 | 704 |
