# Supplementary material for: Changes in the Microbiome in the Soil of an American Ginseng Continuous Plantation
Source: Front Plant Sci. 2020 Dec 7;11:572199. doi: 10.3389/fpls.2020.572199 (PMC7750500; doi:10.3389/fpls.2020.572199)
Supplement: Supplementary Figure 1 — Number of sequences plotted against the coverage of OTUs; each line is standard for one of the 31 samples. [file Data_Sheet_1.zip › Table 3 - 2020-12-02T125434.446.DOCX]

**TABLE S2** Fungal relative abundance in American ginseng cropped soil for 0-4 years.

| **Fungal name** | **LZ1** | **LZ2** | **LZ3** | **LZ4** | **LZCK** |
| --- | --- | --- | --- | --- | --- |
| *Alternaria* spp. | 0.45211 | 0.19618 | 0.23415 | 0.07369 | 0.06523 |
| *Cladorrhinum* spp. | 0 | 0.01828 | 0.01687 | 0.03975 | 0 |
| *Cladosporium delicatulum* | 0 | 0 | 0.01641 | 0.02859 | 0.11509 |
| *Coprinellus bisporus* | 0 | 0 | 0 | 0 | 0.01571 |
| *Cystofilobasidium macerans* | 0 | 0.04261 | 0 | 0 | 0.05501 |
| *Epicoccum nigrum* | 0 | 0.07124 | 0 | 0 | 0 |
| *Exophiala equina* | 0.03408 | 0.04028 | 0.07306 | 0.10756 | 0.12644 |
| *Fusarium* spp. | 0.01358 | 0.02022 | 0.02239 | 0.03158 | 0 |
| *Gibberella baccata* | 0.03014 | 0.02925 | 0.0242 | 0.01695 | 0.02386 |
| *Guehomyces pullulans* | 0 | 0 | 0.06062 | 0.10526 | 0 |
| *Ilyonectria macrodidyma* | 0 | 0.02700 | 0.06662 | 0.06987 | 0.05819 |
| *Minimedusa polyspora* | 0.01348 | 0 | 0 | 0 | 0.01596 |
| *Mortierella* spp. | 0.24595 | 0.35058 | 0.30037 | 0.30408 | 0.22235 |
| *Mrakia aquatica* | 0.02343 | 0.02421 | 0 | 0.01645 | 0 |
| *Paraphaeosphaeria* spp. | 0 | 0 | 0 | 0.02187 | 0 |
| *Paraphoma chrysanthemicola* | 0.02181 | 0.02083 | 0.03601 | 0.02219 | 0 |
| *Penicillium neocrassum* | 0 | 0.000531 | 0 | 0 | 0.012537 |
| *Phlebiella* spp. | 0.16544 | 0.02824 | 0.08023 | 0 | 0.05351 |
| *Pseudaleuria* spp. | 0 | 0.01590 | 0 | 0 | 0.01848 |
| *Sclerotiniaceae* spp. | 0 | 0 | 0 | 0 | 0.04397 |
| *Solicoccozyma* spp. | 0 | 0.03473 | 0.01495 | 0.09284 | 0.02242 |
| *Tetracladium marchalianum* | 0 | 0.01815 | 0 | 0 | 0 |
| *Trichocladium opacum* | 0 | 0 | 0.01924 | 0.02341 | 0 |
| *Vishniacozyma victoriae* | 0 | 0 | 0 | 0 | 0.02658 |
| Other | 0 | 0.06232 | 0.03489 | 0.04592 | 0.13721 |
