# Supplementary material for: Changes in the Microbiome in the Soil of an American Ginseng Continuous Plantation
Source: Front Plant Sci. 2020 Dec 7;11:572199. doi: 10.3389/fpls.2020.572199 (PMC7750500; doi:10.3389/fpls.2020.572199)
Supplement: Supplementary Figure 1 — Number of sequences plotted against the coverage of OTUs; each line is standard for one of the 31 samples. [file Data_Sheet_1.zip › Table 4 - 2020-12-02T125505.843.DOCX]

**TABLE S3** Relative abundance of fungi in the soil planted and non-planted American ginseng at Yinpan and Miaotaizi.

| **Fungal name** | **LZA2** | **LZA2CK** | **LZA3** | **LZA3CK** |
| --- | --- | --- | --- | --- |
| *Boeremia exigua* | 0 | 0.03625 | 0 | 0.02311 |
| *Cadophora* spp. | 0 | 0 | 0.01528 | 0 |
| *Cercophora* spp. | 0 | 0.01554 | 0 | 0 |
| *Clonostachys rosea* | 0 | 0 | 0.01557 | 0 |
| *Corynespora cassiicola* | 0 | 0.07236 | 0 | 0 |
| *Cylindrocarpon* spp. | 0.01978 | 0 | 0 | 0 |
| *Entoloma graphitipes* f. *cystidiatum* | 0 | 0.05354 | 0 | 0 |
| *Epicoccum nigrum* | 0.03406 | 0.04522 | 0 | 0 |
| *Exophiala equina* | 0 | 0 | 0.01606 | 0 |
| *Fusarium* spp. | 0.07157 | 0.01887 | 0.31777 | 0.11470 |
| *Gibberella baccata* | 0 | 0 | 0 | 0.02136 |
| *Gibberella intricans* | 0.01738 | 0.12244 | 0 | 0.02834 |
| *Guehomyces pullulans* | 0.01558 | 0.01637 | 0.01448 | 0 |
| *Ilyonectria macrodidyma* | 0.0256 | 0.01997 | 0 | 0 |
| *Lectera colletotrichoides* | 0 | 0.115718 | 0 | 0 |
| *Microdochium* spp. | 0.01552 | 0 | 0 | 0.02527 |
| *Mortierella* spp. | 0.52584 | 0.28988 | 0.24639 | 0.49066 |
| *Olpidium brassicae* | 0 | 0.02219 | 0 | 0 |
| *Phaeosphaeria fuckelii* | 0 | 0 | 0 | 0.08936 |
| *Plectosphaerella cucumerina* | 0 | 0.02016 | 0 | 0 |
| *Sarocladium* spp. | 0 | 0 | 0.03648 | 0 |
| *Solicoccozyma aeria* | 0.06219 | 0 | 0 | 0.03144 |
| *Tetracladium breve* | 0.02574 | 0 | 0 | 0.02373 |
| *Thelebolus globosus* | 0.02082 | 0.02027 | 0 | 0 |
| *Vishniacozyma heimaeyensis* | 0 | 0 | 0.01373 | 0 |
| other | 0.16591 | 0.13123 | 0.32425 | 0.15204 |
