# Supplementary material for: Changes in the Microbiome in the Soil of an American Ginseng Continuous Plantation
Source: Front Plant Sci. 2020 Dec 7;11:572199. doi: 10.3389/fpls.2020.572199 (PMC7750500; doi:10.3389/fpls.2020.572199)
Supplement: Supplementary Figure 1 — Number of sequences plotted against the coverage of OTUs; each line is standard for one of the 31 samples. [file Data_Sheet_1.zip › Table 5 (76).DOCX]

**TABLE S4** The OTUs number with taxon for bacteria in the soil of different American ginseng cropping years.

| **Bacteria name** | **OTUs number** | | | | | |
| --- | --- | --- | --- | --- | --- | --- |
|  | LZ4 | LZCK | LZA2 | LZA2CK | LZA3 | LZA3CK |
| *A17* | 342 | 194 | 239 | 137 | 937 | 153 |
| *Adhaeribacter* | 143 | 96 | 130 | 873 | 1 | 366 |
| *Aquicella* | 1206 | 452 | 491 | 237 | 174 | 391 |
| *Arthrobacter* | 1093 | 814 | 793 | 1086 | 1308 | 583 |
| *Bacillus* | 1600 | 442 | 1557 | 1264 | 1492 | 457 |
| *Bradyrhizobium* | 1166 | 1248 | 569 | 450 | 1034 | 706 |
| *Burkholderia* | 138 | 360 | 14 | 19 | 415 | 77 |
| *Candidatus_Koribacter* | 1618 | 1782 | 145 | 54 | 3567 | 154 |
| *Candidatus_Solibacter* | 1373 | 1570 | 314 | 116 | 714 | 411 |
| *Candidatus_Xiphinematobacter* | 180 | 194 | 294 | 166 | 26 | 41 |
| *DA101* | 14694 | 7557 | 2358 | 785 | 1708 | 1401 |
| *Dyella* | 254 | 16 | 15 | 2 | 222 | 28 |
| *Flavisolibacter* | 823 | 1022 | 675 | 1148 | 31 | 387 |
| *Flavobacterium* | 719 | 2320 | 1573 | 422 | 529 | 2396 |
| *Hyphomicrobium* | 1268 | 444 | 813 | 393 | 775 | 495 |
| *Kaistobacter* | 9979 | 4472 | 9104 | 5655 | 5352 | 1540 |
| *Luteibacter* | 111 | 44 | 1125 | 10 | 955 | 11 |
| *Luteimonas* | 1402 | 662 | 1619 | 157 | 133 | 104 |
| *Massilia* | 2346 | 2942 | 301 | 1443 | 195 | 636 |
| *Methylibium* | 456 | 491 | 425 | 496 | 156 | 773 |
| *Methylotenera* | 83 | 219 | 1101 | 6480 | 3329 | 1625 |
| *Mycobacterium* | 519 | 302 | 350 | 111 | 334 | 154 |
| *Mycoplana* | 65 | 8 | 50 | 29 | 106 | 106 |
| *Nitrospira* | 931 | 825 | 1323 | 705 | 263 | 1723 |
| *Novosphingobium* | 171 | 54 | 27 | 61 | 40 | 20 |
| *Paenibacillus* | 470 | 147 | 538 | 268 | 481 | 280 |
| *Pedobacter* | 1084 | 1604 | 513 | 52 | 407 | 466 |
| *Planctomyces* | 485 | 421 | 647 | 514 | 169 | 509 |
| *Pontibacter* | 88 | 0 | 455 | 227 | 61 | 5 |
| *Pseudomonas* | 475 | 536 | 1398 | 349 | 78 | 2937 |
| *Pythium* | 187 | 69 | 57 | 15 | 55 | 63 |
| *Ramlibacter* | 290 | 794 | 187 | 585 | 61 | 1022 |
| *Rhodanobacter* | 551 | 8 | 162 | 3 | 2511 | 22 |
| *Rhodoplanes* | 6828 | 2139 | 4033 | 1166 | 5072 | 1733 |
| *Rubrivivax* | 264 | 481 | 348 | 619 | 60 | 714 |
| *Salinimicrobium* | 8 | 0 | 13 | 0 | 58 | 0 |
| *Skermanella* | 222 | 95 | 221 | 651 | 4 | 386 |
| *Sphingobium* | 480 | 21 | 190 | 24 | 24 | 19 |
| *Sphingomonas* | 398 | 736 | 365 | 458 | 184 | 214 |
| *Sporosarcina* | 483 | 118 | 521 | 502 | 600 | 177 |
| *Steroidobacter* | 559 | 106 | 751 | 1168 | 53 | 873 |
| *Thermomonas* | 688 | 251 | 458 | 625 | 140 | 246 |
| *Variovorax* | 260 | 523 | 320 | 782 | 26 | 886 |
