# Supplementary figures and images for: Changes in the Microbiome in the Soil of an American Ginseng Continuous Plantation
Source: Front Plant Sci. 2020 Dec 7;11:572199. doi: 10.3389/fpls.2020.572199 (PMC7750500; doi:10.3389/fpls.2020.572199)

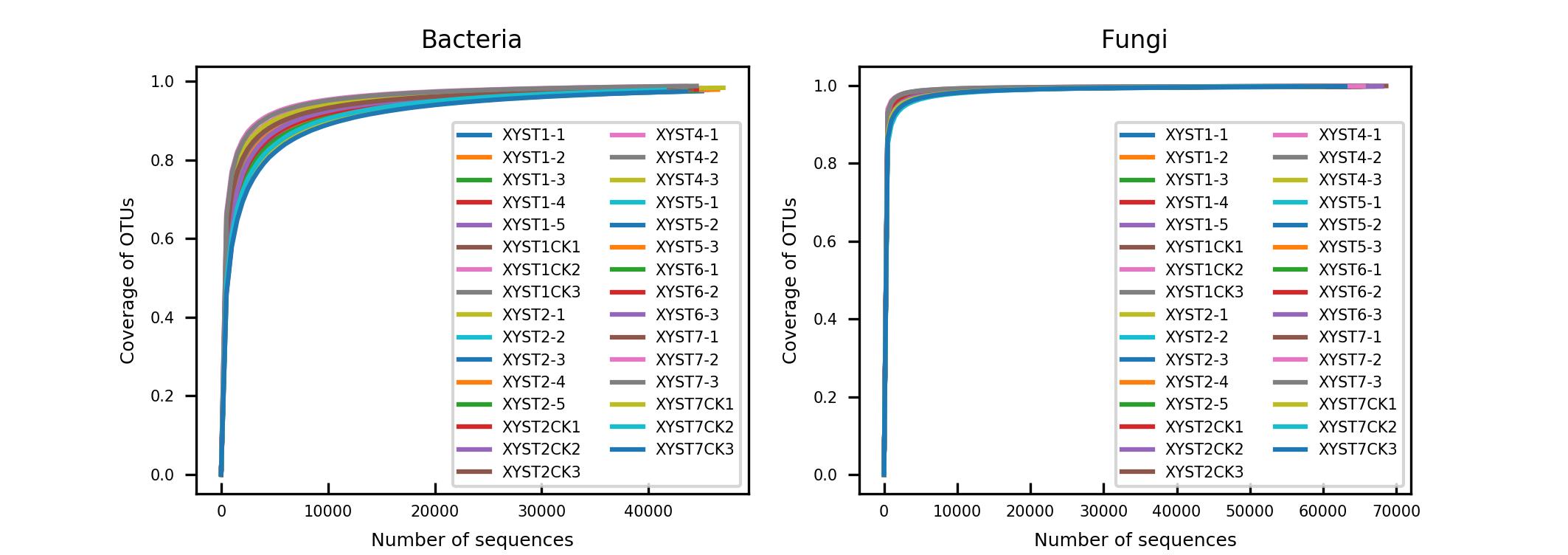

Supplement: Supplementary Figure 1 — Number of sequences plotted against the coverage of OTUs; each line is standard for one of the 31 samples. [file Data_Sheet_1.zip › Image 1 (48).JPEG]
